# Investigation of Cross-Species Translatability of Pharmacological MRI in Awake Nonhuman Primate - A Buprenorphine Challenge Study

**DOI:** 10.1371/journal.pone.0110432

**Published:** 2014-10-22

**Authors:** Stephanie Seah, Abu Bakar Ali Asad, Richard Baumgartner, Dai Feng, Donald S. Williams, Elaine Manigbas, John D. Beaver, Torsten Reese, Brian Henry, Jeffrey L. Evelhoch, Chih-Liang Chin

**Affiliations:** 1 Imaging, Merck & Co. Inc., West Point, Pennsylvania, United States of America; 2 Translational Medicine Research Centre, MSD, Singapore, Singapore; 3 Biostatistics and Research Decision Sciences, Merck & Co. Inc., Rahway, New Jersey, United States of America; 4 Imaging, Maccine Pte Ltd, Singapore, Singapore; Italian Institute of Technology, Italy

## Abstract

**Background:**

Pharmacological MRI (phMRI) is a neuroimaging technique where drug-induced hemodynamic responses can represent a pharmacodynamic biomarker to delineate underlying biological consequences of drug actions. In most preclinical studies, animals are anesthetized during image acquisition to minimize movement. However, it has been demonstrated anesthesia could attenuate basal neuronal activity, which can confound interpretation of drug-induced brain activation patterns. Significant efforts have been made to establish awake imaging in rodents and nonhuman primates (NHP). Whilst various platforms have been developed for imaging awake NHP, comparison and validation of phMRI data as translational biomarkers across species remain to be explored.

**Methodology:**

We have established an awake NHP imaging model that encompasses comprehensive acclimation procedures with a dedicated animal restrainer. Using a cerebral blood volume (CBV)-based phMRI approach, we have determined differential responses of brain activation elicited by the systemic administration of buprenorphine (0.03 mg/kg i.v.), a partial µ-opioid receptor agonist, in the same animal under awake and anesthetized conditions. Additionally, region-of-interest analyses were performed to determine regional drug-induced CBV time-course data and corresponding area-under-curve (AUC) values from brain areas with high density of µ-opioid receptors.

**Principal Findings:**

In awake NHPs, group-level analyses revealed buprenorphine significantly activated brain regions including, thalamus, striatum, frontal and cingulate cortices (paired *t*-test, versus saline vehicle, p<0.05, *n* = 4). This observation is strikingly consistent with µ-opioid receptor distribution depicted by [6-O-[^11^C]methyl]buprenorphine ([^11^C]BPN) positron emission tomography imaging study in baboons. Furthermore, our findings are consistent with previous buprenorphine phMRI studies in humans and conscious rats which collectively demonstrate the cross-species translatability of awake imaging. Conversely, no significant change in activated brain regions was found in the same animals imaged under the anesthetized condition.

**Conclusions:**

Our data highlight the utility and importance of awake NHP imaging as a translational imaging biomarker for drug research.

## Introduction

Pharmacological MRI (phMRI) is a neuroimaging technique that allows characterization of changes in neural activity following drug challenge. Drug-induced pharmacodynamic responses detected from alterations in blood oxygenation level dependence (BOLD) signals or relative cerebral blood volume/flow (rCBV/rCBF) can provide as imaging biomarkers to delineate the central effect of drug actions [1], [2], [3], [4]. As a result, significant effort has been devoted to implement phMRI as a translational research tool to develop novel CNS compounds, considering that this *in vivo* imaging technique can be performed in both animals [5], [6], [7], [8] and humans [9], [10], [11], [12]. To date, phMRI has been adopted to interrogate several key neurotransmitter systems, and these findings collectively have highlighted the value of phMRI to elucidate neurobiological mechanisms of drug actions and to gain better understanding of human diseases. In most preclinical imaging studies, animals were anesthetized during image acquisition to minimize movements; however, several lines of evidence have shown that the use of anesthesia could attenuate basal neuronal activity and likely confound drug-induced brain activation patterns [13], [14], [15], [16], [17], [18], [19], [20]. Therefore, it is critical to establish translational platforms that permit investigation of pharmacological responses of experimental therapeutics in preclinical species, healthy volunteers and eventually in patients. To this end, phMRI in awake animals has been extensively pursued across various species, such as rodents [19], [21], [22], [23] and nonhuman primates (NHPs) [17], [18], [24], [25], [26], [27], [28].

Awake NHP models are particularly attractive for neuroscience research, in light of greater similarities with humans in clinical and pathological characteristics [29]. It has been demonstrated that NHP and human brains share features in neuronal cytoarchitecture [30] and functional connections [31]. Additionally, the activity and distribution of neurotransmitter systems [32], [33], drug metabolism and pharmacokinetic responses [34] are more comparable to human in NHP than in rodent. Nonetheless, despite the improved cross-species translatability offered by awake NHP imaging, animal movements during image acquisition can pose as a major obstacle, since excessive motion can lead to image blurring or ghosting artifacts, and higher baseline signal fluctuations that could diminish sensitivity to detect changes of drug-induced phMRI signals. Furthermore, the level of resting state stability could vary significantly among imaging sessions, which can attribute to compromised test-retest reliability and hindrance to conducting longitudinal studies. To circumvent this challenge, several groups have attempted to develop awake NHP imaging paradigms, in which extensive acclimation procedures are often required [17], [27], [35], [36], [37]. Animals were trained to remain still and gradually habituate to the actual scanning environment while using a dedicated animal restrainer that affords better securing of the animal's head and/or body, such as a head-post model [35] or non-invasive helmet approaches [27], [36]. In addition, improved data acquisition methods [37], use of contrast agent [38] and optimized data analysis strategies [39] have also been explored to enhance phMRI sensitivity. Nonetheless, it will be essential to establish a systematic approach to evaluate phMRI baseline signals attributable to animal movements and thus characterize the utility, or limitation, of these awake imaging platforms.

Buprenorphine is an opioid agonist that has a high affinity to various opioid receptors, such as µ, δ, κ subtypes [40], and it has been used clinically to treat opiate addiction or pain [41], [42]. The clinical efficacy of buprenorphine for pain and opiate addiction is thought to be due to partial agonism at µ–opioid receptors and/or antagonism at κ–opioid receptors [41], [42]. Previous studies performed by Upadhyay et al. have shown that buprenorphine elicited BOLD responses in humans correspond to brain regions with abundant µ–opioid receptors and modulate brain functional connectivity ascribed to pain-processing circuitry [43]. Interestingly, congruency in the spatial pattern of buprenorphine-induced brain activation between humans and conscious rats was also observed [21]. In light of these studies in both humans and awake rats, a phMRI study of buprenorphine in awake NHP can assess the congruency of imaging endpoints across species, and thereby determine the utility of an awake NHP imaging platform for translational research.

In this study, we sought to investigate differential responses in brain activity in NHP produced by the systemic administration of buprenorphine under awake and anesthetized conditions. We hypothesized that buprenorphine-induced brain activation pattern observed from awake NHP should highlight the known µ-opioid receptor distribution [44], [45] and afford investigation of cross-species translatability of awake NHP imaging in conjunction with previous phMRI data obtained from humans and awake rats [21], [43].

Toward this goal, we first constructed a dedicated animal restrainer based on a design previously reported by Andersen et al. [35] and then developed an awake NHP training protocol. Each animal received comprehensive training, including incremental sessions in a mock MRI scanner and acclimatization to the actual imaging environment. The effectiveness of training was evaluated via measured circulating cortisol levels and behavioral scores (e.g. rated by the trainer). Consequently, these criteria were used to select animals thought to be most habituated to the scanner environment for subsequent imaging studies. During the imaging phase, resting state BOLD signals were acquired under awake (both test and re-test) and anesthetized conditions to determine resting state baseline stability and reliability, which served as a measure of the effectiveness or limitation of our acclimation procedures. To assess the cross-species translatability of our awake NHP platform, we imaged brain activity induced by buprenorphine infusion (0.03 mg/kg i.v.) using a CBV-based phMRI approach, where an ultrasmall superparamagnetic iron oxide (USPIO) contrast agent was applied to increase sensitivity [46]. Imaging experiments were conducted following a within-subject study design to differentiate the central effect of buprenorphine and saline vehicle in NHPs imaged under both awake and isoflurane-anesthetized conditions. To characterize the regional specificity of drug effect, a group-level and region-of-interest (ROI) data analysis pipeline was implemented based on a standard monkey brain atlas [47], where drug-induced CBV-based signal change time-course data and corresponding area-under-curve (AUC) were derived from selected brain regions with high density of µ–opioid receptors.

## Materials and Methods

### Ethics Statement

The following experiments were conducted in accordance with the Institutional Animal Care and Use Committee at Merck & Co. The current study, in addition to its protocol, was approved by the Institutional Animal Care and Use Committee at Merck & Co (Permit Number: 11102130610016). No animals were sacrificed for the purpose of this experiment. Trained veterinarians and animal technicians were involved in the care of the animals as well as all the animal procedures performed during this study.

### Animals

Adult female cynomolgus monkeys (Macaca fascicularis, PT Prestasi Fauna Nusantara, Indonesia; *n* = 12, age  = 4 years - 6 years, body weight  = 2 kg∼4 kg) were allocated for this study. Animals were pair housed at the Maccine facility in temperature- (18 °C–26 °C) and humidity-controlled (30%–70%) rooms maintained on a 12∶12 light/dark cycle with lights on at 7:00 AM. Mirrors were placed outside the home cage, and cage toys/manipulative enrichment, such as Kong toys, Flexi keys, were provided and rotated every week. Radios and televisions were also used as supplementary enrichments. Animals were fed with a diet of monkey chow free of animal protein, as well as a controlled amount of fruits or vegetables, offered twice daily. Aside from daily fruit rations, frozen homemade treats (i.e. fruits, raisins, cereals, etc.) were provided once a week. Tap water was offered ad libitum. Prior to the commencement of the study, a complete physical examination, including hematological/blood chemistry analysis, was performed and reviewed by the attending veterinarian.

### Awake Training Protocol

**Figure 1:**
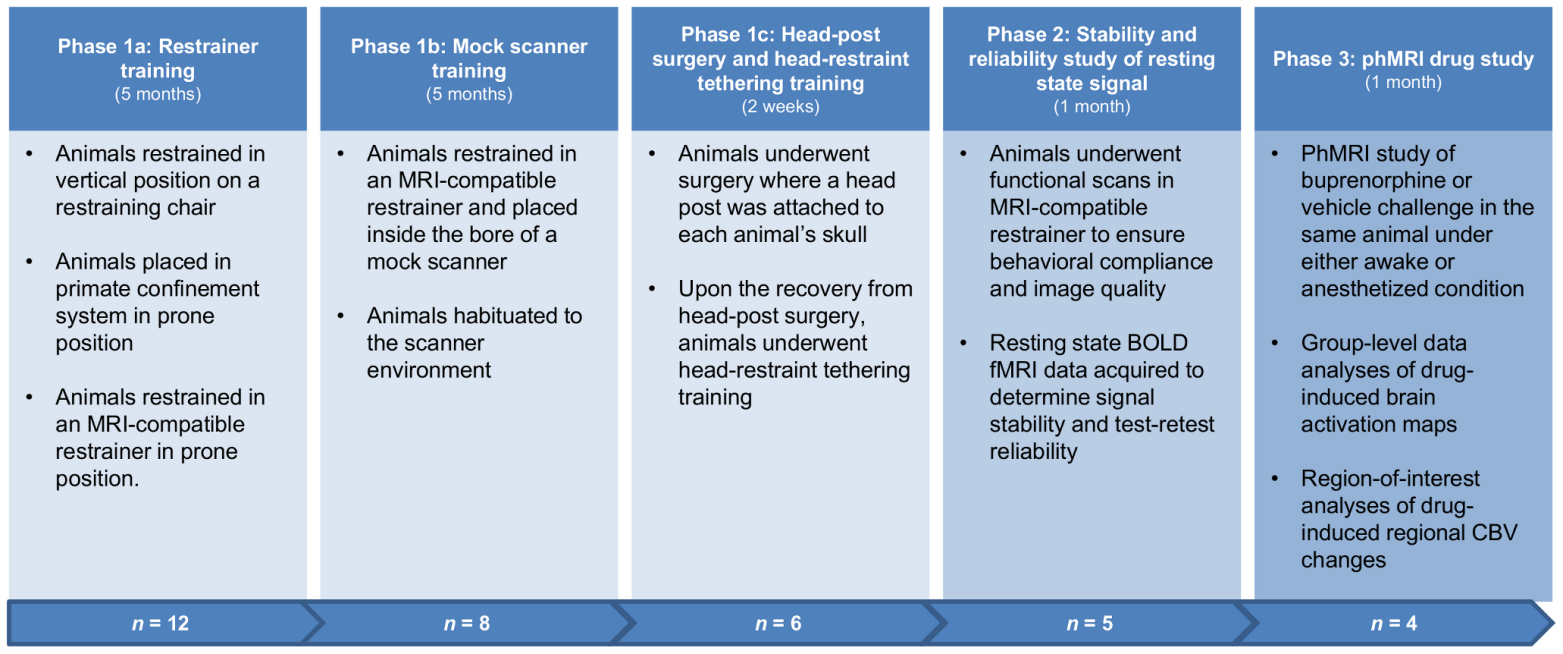
Study protocol of awake imaging in non-human primates. Initial Phase 1a–c focus on the training procedures to reduce animal movement/stress during awake imaging; Phase 2 was the assessment of the stability of resting state signal and its test-retest reliability; Phase 3 was the within-subject pharmacological challenge study, where buprenorphine (0.03 mg/kg i.v.) and vehicle were given to the same animals under awake and anesthetized conditions.

Based on previous work done by Andersen et al. [35], a dedicated animal restrainer was constructed, and the animals were habituated to the restrainer and MRI scanner environment for awake imaging. To alleviate stress and minimize movement during awake imaging, each animal was blindfolded and trained in a phased manner, gradually habituated through different training procedures over several months (see Fig. 1; Phase 1a–c). As shown in Figure 1, our training protocol includes *restrainer training*, *mock scanner training* and *head-post surgery and head-restraint tethering training* phases. During each training session, animal's behavioral gestures (e.g. vocalization, excessive movement, head/body turn) were monitored and scored by a trainer. A low cumulative behavioral score at the end of the training was indicative of a well-behaved animal, suitable for the imaging study. Briefly, during Phase 1a: *restrainer training*, animals were trained to remain still in a primate restraining chair for a period of 30 minutes which was gradually prolonged to a steady state of 120 minutes. Upon achieving this requirement, animals were placed in a prone position in a confinement apparatus for 120 minutes to acclimatize to the restrainer environment. Next, animals were gradually exposed to an MRI-compatible restrainer and the duration of the training period increased by 15 minutes weekly until a steady state of 120 minutes was reached. Additionally, to assess the stress level associated with training procedures, cortisol concentrations were also measured using an electrochemiluminescence immunoassay toolkit (ECLIA, Roche Diagnostics, Singapore), in which blood samples (2 mL) were collected prior to placing the animals inside the restrainer tube and 90-minute-post training. Based on the measured cortisol levels and cumulative behavioral scores over the course of Phase 1a, eight animals were selected and progressed to Phase 1b (*mock scanner training*).

In the *mock scanner training* phase, animals were placed in the restrainer in a mock scanner for a 2-hour time period every other day, in order to habituate them to the scanner environment. At the last training session of this phase, cortisol measurements were repeated (pre- and post-training, *n* = 8) to evaluate the effectiveness of acclimation, and the results were compared with a non-trained control cohort (*n* = 6). Finally, in the *head-post surgery and head-restraint tethering* phase for the selected animals, the head-post component of the restrainer was surgically attached to the skull of each subject [35]. Head tethering restraint training started after animals had fully recovered from the surgery and occurred twice a week until the actual imaging study.

### Animal Preparation for the Imaging Study

Animals progressed from the training procedures to imaging studies (see Fig. 1), where they were imaged under both awake and anesthetized conditions for the stability and test-retest reliability study of resting state BOLD signal and subsequently the phMRI experiments. For the anesthetized imaging study, animals were sedated with ketamine (10 mg/kg IM) for induction, while ear plugs and eye lubricant were applied to all the animals, prior to bringing them into the scanner room. The animal was then placed on the patient bed of the MRI scanner in a prone position and ventilated by an MRI-compatible ventilator and bellows system (SurgiVet, Dublin, OH). Anesthesia was maintained through inhalation of a mixture of isoflurane (2%) in medical air. Physiological parameters of each animal, including heart rate, SpO_2_, EtCO_2_, and respiration rate were monitored and recorded throughout the imaging session using an MR-compatible Datex-Ohmeda physiological monitoring system (GE Healthcare, WI). The temperature of the animal was measured via a fiber-optic temperature sensor system (OpSens, Quebec, Canada) and recorded periodically throughout the experiment. For the awake imaging study, animals were blindfolded and placed in a sphinx position within the restrainer used during the training procedures. A customized head radiofrequency (RF) coil was securely attached to the head-post of the animal. Also, during each awake imaging session, at least one animal trainer would be assigned to visually monitor the animals.

### Evaluation of the Effectiveness or Limitation of Awake Training Protocol

All imaging experiments were conducted on a 3 Tesla TIM Trio MRI scanner (Siemens Medical Solutions, Erlangen, Germany) using a dedicated 8-channel phased-array head coil (RAPID Biomedical GmbH, Germany). Resting state BOLD signal was collected by a single-shot gradient-echo EPI pulse sequence (TR/TE  = 3 s/21 ms, in-plane pixel size  = 1 mm ×1 mm, slice thickness  = 2 mm, and 24 slices with no gap), and 810 imaging volumes (40 minutes in total) were acquired. High resolution brain anatomical images (TR/TE  = 700 ms/13 ms, in-plane pixel size  = 0.67 mm ×0.67 mm) of individual animals were also acquired in order to co-register with a standard monkey brain atlas [47] for further group-level and ROI analyses. Resting state BOLD signals were acquired from the same animals imaged under anesthetized and awake conditions separately (*n* = 5). Further, to examine the test-retest reliability of resting state BOLD signal during awake imaging, experiments were repeated on a different day. As a result, each animal was imaged three times in total.

### Assessment of Cross-species Translatability of Awake NHP

For the phMRI study, selected animals (*n* = 4) underwent four separate sessions (1-week apart to ensure drug washout) on different days (16 scans in total), where a counterbalanced measures design was used to ensure each animal was imaged under different drug challenge (vehicle or buprenorphine) or imaging condition (awake or anesthetized). For each imaging experiment, brain anatomical image and functional datasets were acquired using the same imaging pulse sequences and parameters described above (see *Evaluation of the Effectiveness or Limitation of Awake Training Protocol* section).

To increase the sensitivity of phMRI signals [46], a CBV-based approach using a USPIO contrast agent, 7.5 mg/kg i.v. of Ferumoxytol (AMAG Pharmaceutical, Cambridge MA), was used. A tail vein line was cannulated for the administration of contrast agent and drug, while the indwelling catheter was attached to a saline-filled syringe placed on an infusion pump (Harvard Apparatus, Holliston, MA) located outside the scanner room. All animals were administered intravenously with either 0.03 mg/kg of buprenorphine (Reckitt Benckiser Pharmaceuticals, UK) or saline vehicle. Intramuscular injection of buprenorphine at 0.01 mg/kg or 0.03 mg/kg has been commonly used for analgesia in nonhuman primates [48] and we have adopted the higher dose in the current study. Notably, different doses of buprenorphine were selected in previous phMRI studies in rodent (0.04 mg/kg and 0.1 mg/kg i.v.) or human (0.2 mg/70 kg i.v.) [21], [43]. The following imaging protocol was carried out: (i) 5-minute pre-contrast data acquisition, (ii) bolus injection of the contrast agent via the tail vein line (total injection volume  = 2 mL), (iii) 10-minute pre-drug baseline acquisition (post-contrast), (iv) infusion of either buprenorphine at 0.03 mg/kg i.v. or saline vehicle (total injection volume  = 3 mL) was split into three infusion periods (30 s each) separated by a 1.5-minute interval, in order to minimize potential respiratory suppression related to the buprenorphine injection [43]. Finally, (v) 20-minute period of post-drug imaging acquisition.

### Data Analysis

Data analyses were conducted using the FMRIB Software Library (FSL) (http://www.fmrib.ox.au.ul/fls) and in-house Matlab (MathWorks, Natick, MA). First, voxels of the brain were extracted using a brain extraction tool (FSL BET). To characterize the fluctuation of resting state BOLD signals obtained from awake and anesthetized imaging experiments, time-course data of each voxel was derived and expressed as a percentage change relative to the average signal intensity over the entire imaging time period. Then, for each voxel the mean percentage change was calculated by averaging the absolute value of percentage change over time. Consequently, the mean value and standard deviation over the entire brain volume were calculated and reported. Finally, a Bland-Altman plot was used to assess test-retest reliability of awake resting state BOLD signal.

For the phMRI data analysis, FSL was used. Specifically, our data analysis pipeline included the following steps: motion correction (FSL MCFLIRT), brain extraction, spatial smoothing (FWHM  = 2 mm) and co-registration to a standard monkey brain atlas [47]. To depict activated brain regions, time-course relative CBV change, ΔrCBV(*t*), was first calculated from the raw data using a known relationship [46], [49],



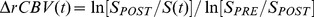
(1)where *t* is time after drug injection, *S*(*t*) is the signal intensity after the drug infusion, *S_POST_* is the mean pre-drug baseline signal intensity over the 10-minute period, post contrast agent administration, and *S_PRE_* is the mean signal intensity over the 5-minute period, prior to the administration of contrast agent. Then, a general linear model (GLM) was used for the subject-level analysis, where a linear ramp model was used to mimic the infusion response, while the elimination of buprenorphine was based on the known plasma pharmacokinetic of buprenorphine in dogs (brain half-life ∼4.5 hr) [50]. Due to the relatively long onset of the ramp function compared to the hemodynamic response function (HRF), the ramp stimulus function in our GLM was not convolved with HRF [43]. To perform unbiased univariate linear regression analysis, nuisance regressors were prepared that account for most of the confounding explanatory variables (EVs), where the mean time-course signals extracted from the ventricles and white matter were included to regress out additional confounds related to physiological noises and/or linear drifts. Further, an in-house Matlab script was written to identify excessive intensity changes (head movement) between imaging time-points, as a de-spiking in the EV. Spike detection was implemented using a median absolute residual variation (MARV) score-based approach [51], while imaging volumes with motion greater than half of a voxel were identified as spikes.

For the group-level and ROI analyses, functional data of each animal was first co-registered to the individual's anatomical images by rigid body translations and rotations and subsequently into a template monkey brain atlas [47] using 12 degrees of freedom affine transformation. These calculated transformation matrix parameters were then applied to the functional dataset and derived statistical maps. Group comparisons were conducted using a mixed-effects paired *t*-test (FSL FLAME) to determine the group means of the differential effect of vehicle and buprenorphine in both anesthetized and awake animals, threshold at p<0.05, in order to identify brain regions affected by the drug or vehicle challenge under awake and anesthetized imaging. In addition, to illustrate the temporal dynamics of rCBV change, time-course data for each imaging condition from selected brain regions were extracted, detrended and plotted. Regional time-course data was calculated by averaging the rCBV change over all the supra-threshold voxels within specific brain regions. Results obtained from the vehicle infusion were exploited to detrend buprenorphine data on a region-by-region basis. Also, ROI analyses of drug-induced ΔrCBV time-course data were performed, in which AUC were obtained from brain regions with high density of µ-opioid receptor such as putamen, caudate, thalamus, cingulate cortex and frontal cortex, where regional AUC values (mean ± SEM) were calculated from all animals treated with either buprenorphine or vehicle and imaged under both anesthetized and awake conditions. Finally, calculated AUC data were subjected to a paired *t*-test (JMP 7.0.1, SAS Institute, Cary NC), with differences considered statistically significant at p<0.05.

## Results

### Behavioral Scores and Cortisol Levels for Animal Selection

**Figure 2:**
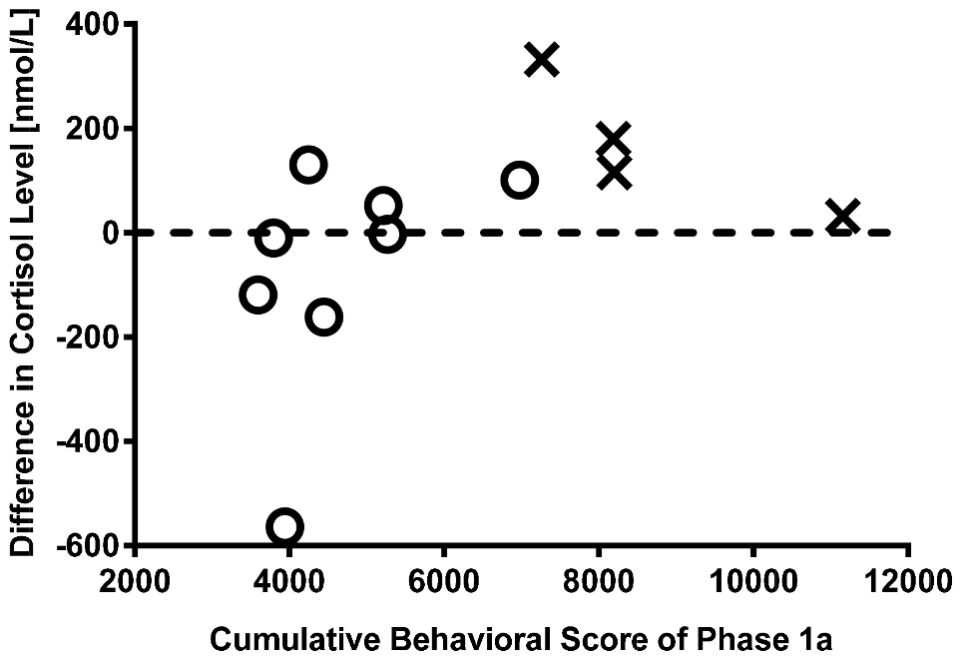
Differences in plasma cortisol concentration and cumulative behavioral scores during Phase 1a used to select animals for Phase 1b. Cumulative behavioral scores over the Phase 1a training obtained from all animals (*n* = 12), in which animals with lower behavioral scores (*n* = 8, ‘O’) were selected to transition into the Phase 1b study and ones with higher scores were deselected (*n* = 4, ‘×’). In addition, to assess the stress level during the awake training, plasma cortisol concentrations were measured from these animals at two separate time points (∼ one month apart) during this phase, and results indicated that the reduction in cortical level is likely associated with better habituation (i.e. lower behavioral scores).

**Figure 3:**
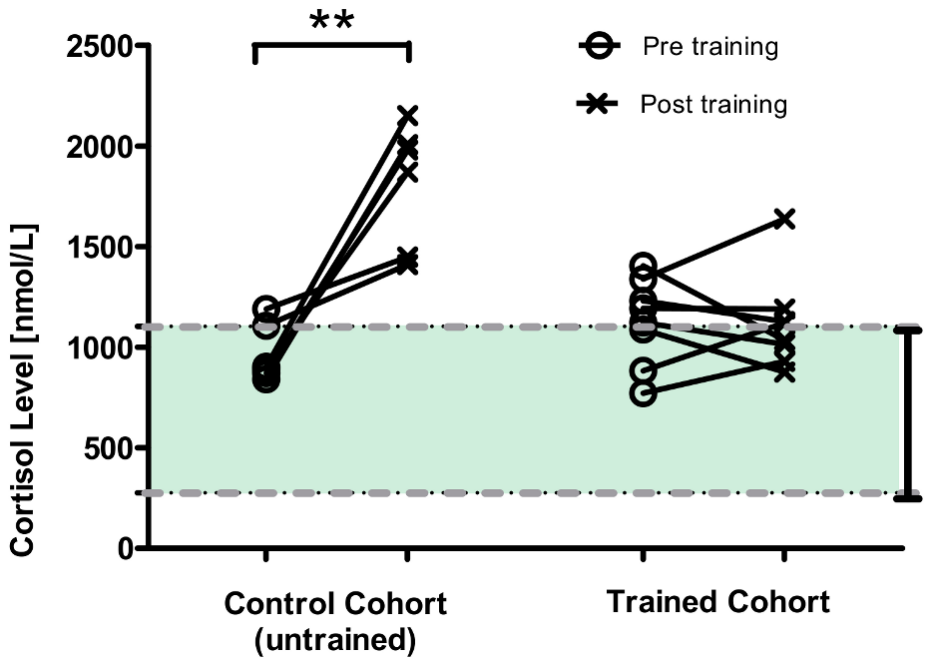
Awake training increased plasma cortisol concentrations in untrained animals, but not in trained animals. Plasma cortisol concentrations measured from the animals (*n* = 8) at prior- and post-90-minute training session during the Phase 1b period. Results indicate that, at the end of training session cortisol levels are significantly elevated in untrained/control animals (*n* = 6), while no significant change was observed in habituated animals (paired *t*-test, **p<0.01). The dash lines highlight the range of normal cortisol concentration observed in cynomolgus monkeys (276 nmol/L to 1104 nmol/L) [52].

Cumulative behavioral scores obtained from all the animals (*n* = 12) over the entire Phase 1a period are plotted in Figure 2, in which animals with lower overall behavioral scores (better habituated) were selected to transition from Phase 1a to 1b (*n* = 8). These results demonstrate that levels of acclimation can vary within the training cohort, and thereby the selection of desired animals is essential. Within this phase, differences in the cortisol levels measured at two time points (separated by a month) are shown in Figure 2, indicating that elevated cortisol levels are likely observed from animals with higher behavioral scores. To assess the effect of training on stress levels, Figure 3 illustrates the cortisol concentrations obtained from untrained/control animals (*n* = 6) as well as our imaging cohort (*n* = 8) at pre- and 90-minute-post training session (Phase 1b). As shown, cortisol levels were significantly elevated in untrained animals after they underwent the training session (paired *t*-test, p<0.01), while no significant difference was observed in habituated animals. Further, only one from the trained imaging cohort had cortisol concentrations substantially above the previously reported normal range (between 276 nmol/L and 1104 nmol/L) [52]. The lower cortisol level found in the trained cohort implies reduced stress levels in these animals and effective habituation to the restrainer and MRI scanner environment. Finally, two animals were deselected from the cohort due to medical conditions (unrelated to the training), while the remaining six animals were moved forward to Phase 1c.

### Effectiveness or Limitation of Awake Training Protocol

**Figure 4:**
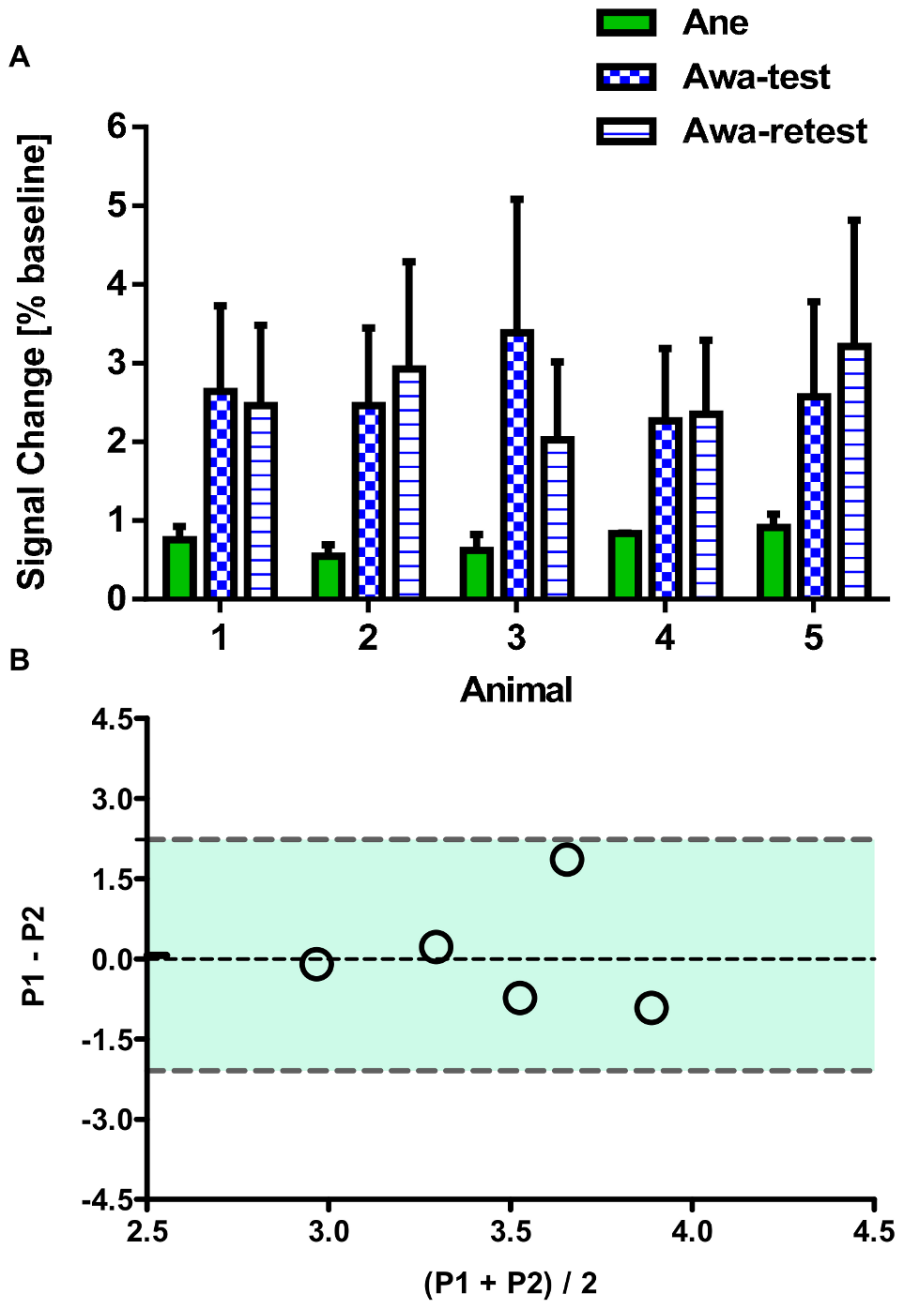
Comparison of resting state stability between anesthetized and awake imaging and test-retest reliability of awake baseline signal. (**A**) Resting state variation of BOLD signal (mean ± SD) obtained from each non-human primate (NHP) imaged under either awake (Awa, test and retest) or anesthetized (Ane) condition. (**B**) Bland-Altman plot for awake imaging data and the dash lines highlight the 95% limits of agreement (P1: represents the first test, P2 represents the retest).

Figure 4A shows the percentage variation in resting state baseline signals within the whole brain volume (mean ± SD) from individual animals imaged under awake and anesthetized conditions. Our data indicate that compared to anesthetized imaging, the level of baseline variation was significantly higher (4∼5 fold) under the awake condition (paired *t*-test, p<0.01). This observation is not unexpected, since there was some animal movement during awake imaging. These findings suggest that a larger change in drug-induced phMRI signals may be needed in order to achieve comparable sensitivity offered by imaging anesthetized animals (i.e. to offset the increased baseline noise). Reasonable test-retest reliability was found from the awake baseline data, considering all the data points were within 95% limits of agreement of the Bland Altman plot (see Fig. 4b). Finally, two animals were deselected from the Phase 3 drug study, due to the increased body weight thus unable to fit into the animal restrainer or the need for repetitive head-post repairs.

### Buprenorphine-induced phMRI response under awake and anaesthetized conditions

**Figure 5:**
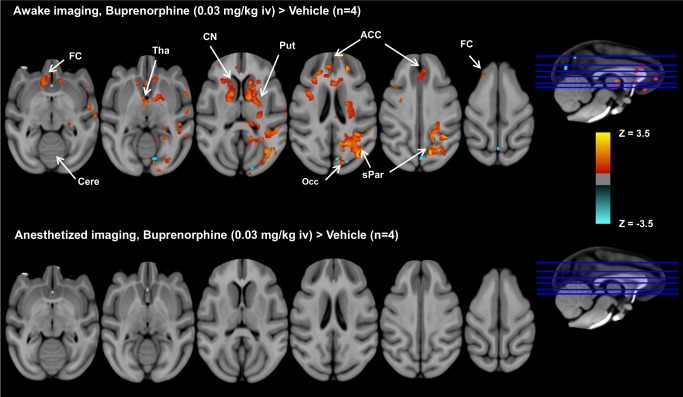
Group comparisons of brain activation patterns between buprenorphine and vehicle under awake and anesthetized imaging. Group comparisons of brain activation patterns showing significant effect of buprenorphine versus vehicle (paired *t*-test, p<0.05, *n* = 4) under awake and anesthetized imaging. In the awake study, buprenorphine activates brain regions with a high density of µ-opioid receptor, including frontal cortex (FC), thalamus (Tha), anterior cingulate cortices (ACC), caudate nucleus (CN), putamen (Put), and superior parietal lobule (sPar), and limited deactivation was found in occipital cortex (Occ). In contrast, no significant difference in brain activation between buprenorphine and vehicle infusion was observed in anesthetized animals.

Figure 5 illustrates group comparisons of brain activation patterns showing significant effect of buprenorphine versus vehicle on rCBV changes (paired t-test, p<0.05, n = 4), while Table 1 lists the results of ROI analyses, including activated brain regions/coordinates, z-statistics, and total percentage activated volumes calculated from the awake study. As shown, under the awake imaging condition, buprenorphine activated brain regions with a high density of µ-opioid receptor including, frontal cortex, thalamus, cingulate cortex, caudate nucleus, putamen, occipital cortex, and superior parietal lobule. Notably, these findings are strikingly consistent with µ-opioid receptor distribution depicted by [6-O-[^11^C]methyl]buprenorphine ([^11^C]BPN) positron emission tomography (PET) study in baboons, where the highest [^11^C]BPN uptakes was observed in striatum (caudate nucleus and putamen) followed by thalamus, cingulate, frontal, parietal, occipital cortices, and then cerebellum [44]. In accordance with the results obtained from the previous study in conscious rodent and human studies [21], similar region-specificity of activated brain structures are also observed in our awake NHP data. Conversely, no significant activation was observed from anesthetized animals (paired *t*-test, vs. vehicle treatment; see Fig. 5B), despite animal physiological parameters (mean ± SEM, *n* = 4) being maintained within normal ranges during these experiments (mean heart rate  =  (119.9±5.1) beats/min, mean SpO_2_  = 98.8%±0.6%, mean EtCO_2_  = 22.0%±0.4%, and body temperature  = 36.5 °C±0.1 °C). These results exemplify the undesired anesthetic-drug interactions embedded in the anesthetized animal experiments, while highlighting the need for awake imaging for phMRI studies.

**Table 1:** Activated brain regions and their volumes induced by buprenorphine infusion derived from the region-of-interest analysis.

Brain Region	Coordinates (X, Y, Z) [mm]	Z-statistics	Activated Volume (percentage) [mm^3^]
Cingulate cortex	29.7, 70.0, 25.2	2.52	182.52 (10.80%)
Gyrus rectus	29.5, 61.2, 21.0	2.61	44.39 (11.58%)
Orbital gyrus	26.0, 58.7, 19.5	2.39	2.83 (0.38%)
Caudate Nucleus	35.5, 57.2, 28.0	2.54	224.19 (28.51%)
Putamen	24.2, 57.2, 20.7	2.43	17.84 (2.02%)
Superior frontal gyrus	17.5, 54.5, 35.5	2.51	77.17 (1.46%)
Middle frontal gyrus	17.2, 54.7, 35.0	2.54	11.14 (0.86%)
Precentral gyrus	51.5, 50.5, 23.5	2.51	43.16 (0.79%)
Postcentral gyrus	53.0, 42.0, 25.7	2.66	34.48 (1.79%)
Globus pallidus	37.0, 50.2, 25.0	2.46	27.55 (13.42%)
Internal capsule	36.2, 49.7, 24.7	2.49	23.22 (10.56%)
Amygdala	40.5, 48.2, 12.7	2.50	13.94 (2.37%)
Thalamus	34.5, 48.2, 27.0	2.61	34.48 (3.18%)
Hippocampus	43.2, 43.0, 12.7	2.61	24.06 (4.84%)
Insular cortex	46.7, 42.7, 21.2	2.51	1.09 (0.28%)
Inferior temporal gyrus	48.0, 40.7, 9.5	2.33	0.25 (0.03%)
Superior temporal gyrus	50.2, 31.7, 29.5	2.53	213.33 (6.32%)
Middle temporal gyrus	50.5, 31.0, 29.7	2.61	43.61 (2.89%)
Midbrain	36.2, 39.7, 18.2	2.37	3.28 (0.34%)
Pulvinar nuclei	36.5, 36.2, 27.0	2.43	6.19 (6.14%)
Inferior parietal lobe	38.0, 24.5, 36.7	2.63	120.39 (4.32%)
Superior parietal lobe	36.2, 21.2, 34.5	2.61	72.75 (2.56%)
Superior occipital	37.7, 22.5, 35.0	2.61	87.05 (2.58%)
Lateral occipital	34.2, 12.0, 27.5	2.56	68.58 (3.16%)
Cerebellum	35.5, 19.0, 22.7	2.43	5.77 (0.08%)

Brain regions show differential responses of buprenorphine infusion (0.03 mg/kg i.v.) in awake imaging (vs vehicle, paired *t*-test, *z*>2.3, *n* = 4). The *z*-statistic represents the maximum of each brain region with significant difference in rCBV changes between buprenorphine and vehicle treatments, while the coordinates correspond to the location of individual maxima in the standard monkey brain atlas space [47].

**Figure 6:**
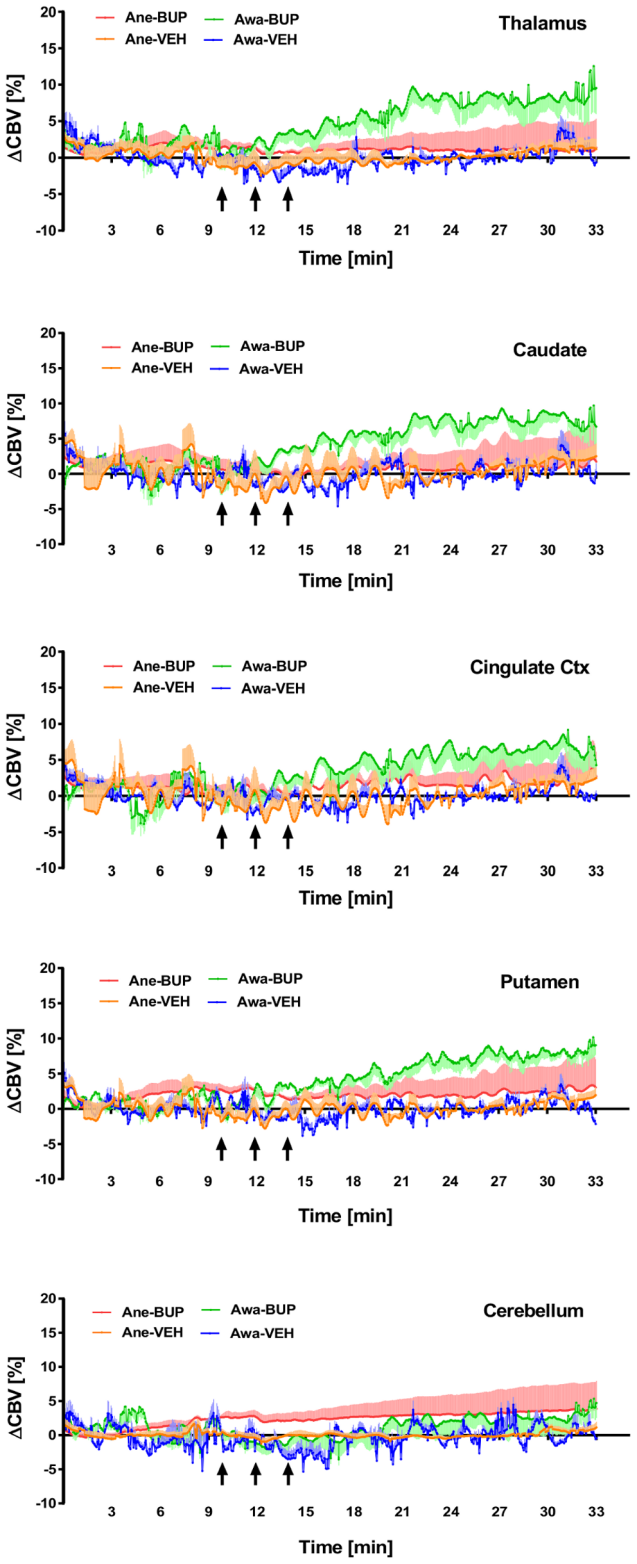
Regional time-course rCBV changes derived from the region-of-interest analysis. Plots of regional time-course rCBV change (mean ± SEM) derived from region-of-interest (ROI) analyses of the imaging cohort (*n* = 4) treated with either buprenorphine (BUP at 0.03 mg/kg i.v.) or vehicle (VEH) and imaged under awake (Awa) or anesthetized (Ane) conditions. The black arrows indicate the three individual dosing time periods (30 s drug infusion separated by 1.5-minute intervals), starting at 10-minute after the contrast agent administration. Results indicated that in awake NHP, buprenorphine infusion produces significant increases in rCBV at brain regions with high density of µ-opioid receptors, but not the area with low concentration of µ-opioid receptors (i.e. cerebellum).

**Figure 7:**
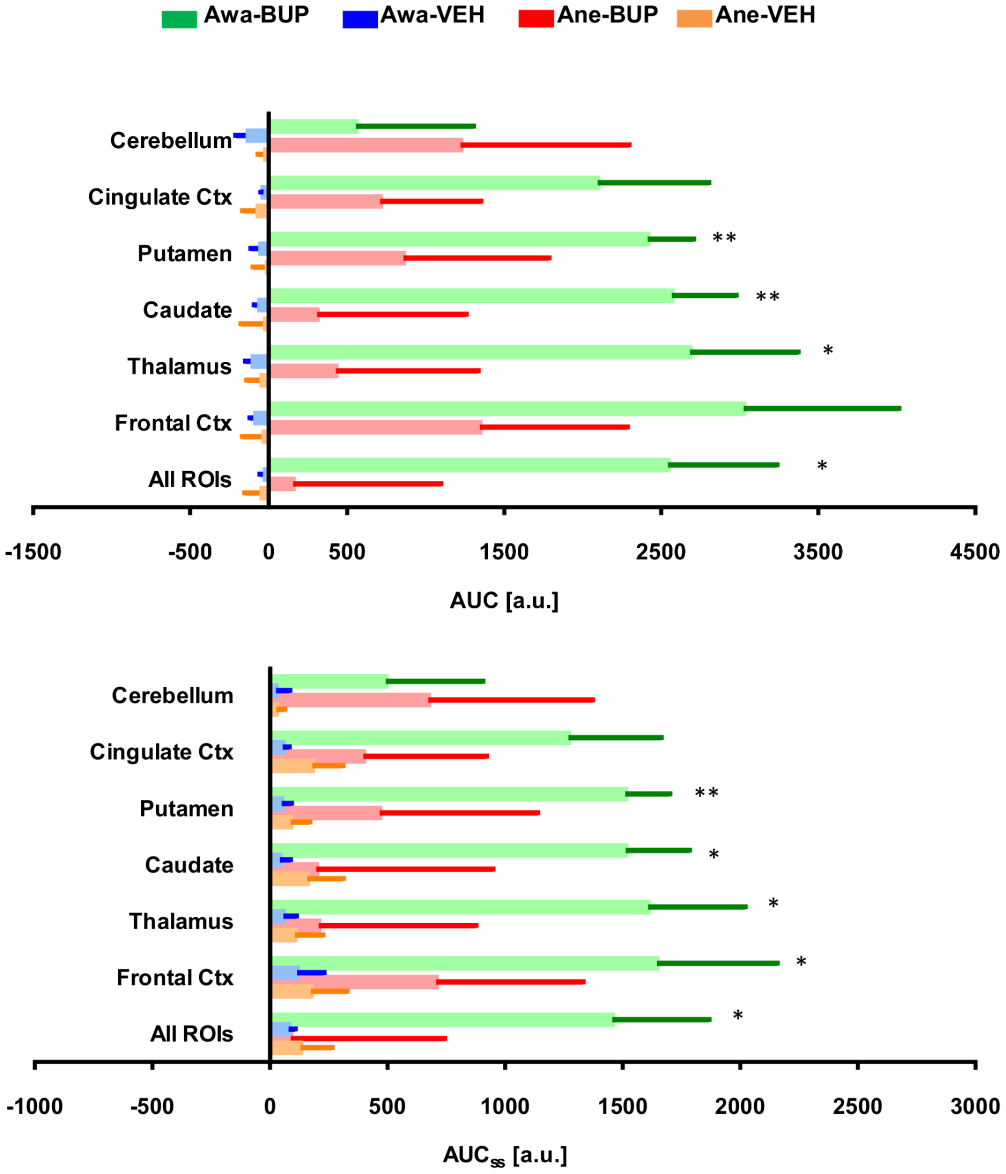
Area-under-curve of regional time-course rCBV change during post-drug infusion derived from the region-of-interest analysis. Results obtained from region-of-interest (ROI) analyses of rCBV time-course data showing the area-under-curve (mean ± SEM) of the entire post-drug infusion period (AUC) or only the steady-state time period (AUC_ss_). Animals (*n* = 4) were imaged under awake (Awa) or anesthetized (Ane) conditions and treated with either buprenorphine (BUP at 0.03 mg/kg i.v.) or vehicle (VEH). Our data indicated that in awake animals AUC and AUC_ss_ calculated from thalamus, caudate nucleus, and putamen are significantly higher in buprenorphine-treated animals (paired *t*-test, *p<0.05, **p<0.01 vs vehicle).

Plots of time-course rCBV changes derived from our ROI analyses are shown in Figure 6, in which activated brain regions identified in the group-level brain activation maps (see Fig. 5) showed significant increases in rCBV following buprenorphine infusion. Buprenorphine produced significant increases in rCBV change (mean ± SD) at frontal cortex (7.3%±0.2%), thalamus (6.5%±0.2%), caudate nucleus (6.2%±0.2%), putamen (5.8%±0.2%), and cingulate cortex (5.1%±0.2%). Of note, it appears that rCBV reached the maximum value around 10-minute-post onset of drug infusion and then maintained at steady state that prolonged over the rest of the imaging period. Area-under-curve of the rCBV time-course data over the entire drug infusion period (AUC), or the above-mentioned steady state period (AUC_ss_), were calculated and shown in Figure 7. Results depicted that both AUC and AUC_ss_ calculated from thalamus, caudate nucleus, and putamen are significantly higher in buprenorphine-treated awake animals (paired *t*-test, p<0.05, vs vehicle), whilst no significant increases in AUC and AUC_ss_ were found in any brain region from the anesthetized study.

## Discussion

Previous PET studies in baboons with the opioid receptor radioligand, [^11^C]BPN revealed high binding activity in striatum, thalamus, cingulate gyrus, frontal cortex, parietal cortex, occipital cortex and cerebellum (in a descending order) [44] and these findings agree well with the known opioid receptor distribution in post-mortem human brain [53]. In this work, we found that buprenorphine infusion in awake, but not anesthetized, NHP produced significant and persistent increase in rCBV at specific brain regions including frontal and cingulate cortices, caudate nucleus, putamen, thalamus, parietal cortex and occipital cortices (see Fig. 5). These activated brain regions are consistent with the binding sites of µ–opioid receptors highlighted in the PET study [44], reflecting the underlying drug actions and subsequent biological responses. Recently, Becerra et al. demonstrated that brain regions activated by buprenorphine in awake rats paralleled that observed in humans, in which buprenorphine elicited BOLD signals in somatosensory and cingulate cortices, insula, striatum, thalamus, periaqueductal gray (PAG), and cerebellum in a dose-dependent manner [21]. Further, in contrast to human data, negative BOLD signals were observed in several brain regions in awake rats and this discrepancy was reconciled with the difference in opioid receptor subtypes and binding affinities between these two species [21]. To examine the congruency of phMRI data across rodent, NHP and humans, it appears that the brain activation patterns obtained from our awake NHP study show greater similarity to human data. For example, very limited deactivation, or decreased rCBV, was found in occipital cortex (see Fig. 5). Also, with the exception of activation in the cerebellum, brain regions activated by buprenorphine in awake NHP are depicted in human data. We attributed inconsistency in cerebellar activation to the difference in drug pharmacokinetic or exposure levels, considering that in the baboon PET study [^11^C]BPN uptake at cerebellum peaked within 2-minute post tracer injection and eliminated rapidly [44]. Further, unlike previous rodent and human BOLD fMRI studies [21], [43], we used a CBV-based approach in the current work. For the CBV-based approach, changes in MRI signal reflect changes in CBV, rather than the concentration of deoxyhemoglobin as in BOLD imaging [54]. While it could depend on the resting state blood volume at specific brain regions, BOLD and CBV contrasts are known to be spatially [54] and temporally [55] coupled. Overall, our data highlight the importance and utility of awake imaging in NHP.

In anesthetized animals, brain activation patterns induced by buprenorphine challenge differ from the previous PET µ–opioid receptor data. In comparison with the vehicle treatment, buprenorphine challenge did not elicit any significant changes in brain activity (see Fig. 5), possibly due to the effect of anesthesia. Also, ROI analyses indicate that the rank order of regional rCBV changes induced by buprenorphine infusion was different between anesthetized and awake imaging (see Fig, 7). For example, in awake NHP the highest rCBV change was found in frontal cortex and followed by thalamus, caudate, putamen, cingulate cortex, and cerebellum, while the rank order (descending) became frontal cortex, cerebellum, putamen, cingulate cortex, thalamus, and caudate under anesthetized imaging. Anesthetic-drug interactions on brain activity has been investigated by several groups [14], [16], [18], [19], and it was concluded that drug-induced brain activation pattern can vary among the anesthetics used. The extent of anesthesia confounds likely pivots on the particular mechanism of action associated with selected anesthetics and test drugs, which might or might not be an issue, depending on the neurotransmitter system investigated. Considering the observed drug-induced activation can result from drug-receptor downstream biological effects, the use of anesthesia can modulate both direct and indirect neuronal pathways. As such, it is more desirable to perform imaging in preclinical species without anesthesia that enables qualification of imaging biomarkers which represent a closer scenario to that of clinical investigation.

One of the key challenges for awake imaging is to ensure animals remain still during the data acquisition. Significant efforts have been made to establish comprehensive habituation procedures and specialized animal restrainers in order to minimize head and body motion during data acquisition [16], [17], [24], [27], [35], [37], [56], [57]. For example, Keliris et al. have developed a sophisticated MRI-compatible training chair and head-post restrainer to image alert monkeys in a natural upright sitting position, and they found jaw and body movements are the major causes for the fluctuation of fMRI signals [56]. Alternatively, Chen et al. have proposed another approach of integrating an MRI-compatible chair, head-post holder, and volume coil for awake imaging, where in-plane movements can be limited within 80 µm [37]. We have adopted the awake imaging apparatus developed by Andersen et al. [35] and used a custom-made NHP phased-array head coil that can be attached to the head-post for better signal-to-noise ratio (SNR). Without a dedicated NHP MRI scanner [37], [56], we have successfully imaged animals in a sphinx position instead. Improved data acquisition and analysis methods have been also developed to enhance the sensitivity to detect targeted drug responses; for example, USPIO imaging contrast agents have been previously exploited to amplify signal changes [36], [38] as well as advanced data analysis pipeline for motion correction [39].

To minimize the amount of stress during awake imaging, our study cohort has been trained over several months to acclimatize to the restrainer and scanner environment (see Fig. 1). Some animals showed signs of gradual adaptation to the training, as indicated by the decreased plasma cortisol concentration and behavioral scores (see Fig. 2). This finding is consistent with the results reported by Lee et al. [58], where a weekly decline in cortisol levels was observed in animals receiving chair training for a month. Cortisol levels were also measured in our study, as animals progressed to Phase 1b. We found no significant difference in cortisol concentrations measured at pre- and post- 90-minute training from the trained cohort, whilst in the untrained animals cortisol concentrations elevated significantly (see Fig 3), suggesting that trained animals have been acclimated to the restrainer.

It is noteworthy that in most awake imaging models, a head-post is surgically implanted for the purpose of head fixation. This invasive procedure is however not ideal since the head-post placement requires surgery and can become loose or even detach over time. While this method might be sub-optimal for a longitudinal study, alternative non-invasive approaches have been recently implemented [27], [36]. For example, Shrihasam and his colleagues have developed a non-invasive vacuum helmet technique for imaging alert monkeys, and they found that the degrees of translational and rotational head motion during awake imaging are comparable to the same monkey's movements when restrained by a head-post [36]. Likewise, using the expandable foam in combination with a plastic helmet, Murnane et al. have shown the feasibility of imaging conscious monkeys under cocaine challenge without a head-post [27]. There are also disadvantages for this awake imaging approach. For example, awake training is time-consuming and often requires significant effort and resources to habituate the animal. For the current study, it took more than 10 months to complete the awake training protocol and only a sub-group of animals was suitable for the actual imaging study. Although the stress level can be alleviated by extensive training procedures, this factor cannot be completely eliminated.

To develop novel treatments for neurological diseases or psychiatric disorders, PET or single photon emission computed tomography (SPECT) imaging techniques have proved to be useful tools to confirm blood-brain barrier penetration and drug target engagement in the brain, and derived receptor occupancy–drug concentration curves can guide dose selection in clinical development [59], [60]. Conversely, biological consequences of drug actions depicted by awake phMRI can offer complimentary pharmacodynamic biomarkers to PET/SPECT imaging. In light of the low success rate associated with the development of novel compounds to treat neurological diseases or psychiatric disorders, phMRI in alert, behaving NHP can serve as a translational imaging platform that affords pharmacodynamic or safety biomarkers, attributed to targeted mechanisms of action. While animal movement and stress during awake imaging increases the noise associated with this approach, the absence of unwanted anesthetic-drug interactions can outweigh efforts needed to overcome these technical hurdles. For the development of CNS compounds, this requirement can be particularly crucial to assess novel experimental therapeutics.

There are limitations for this current study. First, only four animals progressed to the actual imaging experiments and therefore inter-subject variability cannot be properly evaluated. Second, to fully evaluate the utility of this awake phMRI platform, test-retest study of buprenorphine infusion needs to be explored. Third, in the anesthetized imaging study, buprenorphine did not elicit significant changes in rCBV at brain areas with a high density of µ–opioid receptor (see Figure 5). This observation can be attributed to the level of anesthesia used. Previously, Tinker et al. have reported that the percentage volume of minimum alveolar concentration (MAC) of isoflurane in nonhuman primate is 1.28% and the maintaining dose for most applications is around 1.3× MAC (i.e. ∼1.7%) [61], whereas 2% isoflurane in medical air was selected to anesthetize animals in our study. In combination with paralytics (e.g. vercuronium or mivacurium) or fentanyl, the feasibility of using a lower level of isoflurane (0.3%∼1%) in fMRI studies has been demonstrated [62], [63]. However, anesthesia protocols could vary among different studies, depending on the experimental design.

In summary, we have shown that, in awake NHP buprenorphine activated brain regions ascribed to high density of µ-opioid receptors, including caudate nucleus, putamen, thalamus, and cingulate cortex. More importantly, in conjunction with previous buprenorphine phMRI studies in conscious rats and humans, our results establish utility of buprenorphine phMRI across species. Further, based on a standard monkey brain atlas, we have established robust phMRI data and ROI analysis pipeline that allows determination of group-level brain activation patterns. Our results indicated that in the same animal, buprenorphine-induced rCBV changes in awake animals are significantly higher than those obtained under the anesthetized condition. Taken together, our data highlight the utility and importance of awake NHP imaging platform as a translational imaging biomarker for drug research.
